# Ginger (*Zingiber officinale* Roscoe) in the Prevention of Ageing and Degenerative Diseases: Review of Current Evidence

**DOI:** 10.1155/2019/5054395

**Published:** 2019-08-20

**Authors:** Nur Fatin Nabilah Mohd Sahardi, Suzana Makpol

**Affiliations:** Department of Biochemistry, Faculty of Medicine, Level 17, Preclinical Building, Universiti Kebangsaan Malaysia Medical Centre, Jalan Yaacob Latif, Bandar Tun Razak, Cheras, 56000 Kuala Lumpur, Malaysia

## Abstract

Currently, the age of the population is increasing as a result of increased life expectancy. Ageing is defined as the progressive loss of physiological integrity, which can be characterized by functional impairment and high vulnerability to various types of diseases, such as diabetes, hypertension, Alzheimer's disease (AD), Parkinson's disease (PD), and atherosclerosis. Numerous studies have reported that the presence of oxidative stress and inflammation contributes to the development of these diseases. In general, oxidative stress could induce proinflammatory cytokines and reduce cellular antioxidant capacity. Increased oxidative stress levels beyond the production of antioxidant agents cause oxidative damage to biological molecules, including DNA, protein, and carbohydrates, which affects normal cell signalling, cell growth, differentiation, and apoptosis and leads to disease pathogenesis. Since oxidative stress and inflammation contribute to these diseases, ginger (*Zingiber officinale* Roscoe) is one of the potential herbs that can be used to reduce the level of oxidative stress and inflammation. Ginger consists of two major active components, 6-gingerol and 6-shogaol, which are essential for preventing oxidative stress and inflammation. Thus, this paper will review the effects of ginger on ageing and degenerative diseases, including AD, PD, type 2 diabetes mellitus (DM), hypertension, and osteoarthritis.

## 1. Introduction

The term “ageing” has been used to describe the progressive loss of physiological integrity associated with functional impairment and high vulnerability to many types of disease [1]. The elderly population refers to individuals older than 60 years of age. According to the WHO, the proportion of the world's population older than 60 years of age will nearly double from 962 million to 2.1 billion in 2050 [2]. By 2020, the size of this population will exceed the number of children younger than 5 years of age. Moreover, in 2050, 80% of the older population is expected to live in low- and middle-income countries. This increase in the ageing population is due to the high birth rate in 1970 and the decrease in mortality rate between 1970 and 2010 [3]. In 1970, the total fertility rate was 4.95 children per woman, and this rate decreased to 2.1 children per woman in 2010. The mortality rate between 1980 and 2010 steadily decreased from 15.3 to 4.6 per 1000 population.

The process of ageing is heterogeneous and multifactorial and can be caused by biological, social, and physiological factors [4]. A study carried out by López-Otín et al. [1] proposed that the following nine cellular hallmarks are the key attributes of the ageing process: genomic instability, epigenetic alteration, telomere attrition, proteostasis loss, deregulated nutrient sensing, cellular senescence, mitochondrial dysfunction, altered intracellular communication, and stem cell exhaustion. Changes in these cellular hallmarks, which occur more frequently with advancing age, affect the normal function of the cell and lead to the ageing process. Ageing is always related to several forms of disability, such as physical disability, chronic disease, and poor mental functioning [5].

Therefore, ageing is usually associated with degenerative diseases and affects the function and structure of organs or tissues, which deteriorate over time [6]. The deterioration of organ or tissue leads to the gradual decline in physical and mental function and increases the risk of disease, which can result in death. Degenerative disease refers to a disease that occurs when cells and tissues lose their ability to optimally function [7]. Common health problems associated with degenerative diseases are Alzheimer's disease (AD), Parkinson's disease (PD), atherosclerosis, cardiovascular disease, hypertension, and type 2 diabetes mellitus (DM) [7, 8]. Biologically, ageing and degenerative diseases are the consequences of cellular and molecular damage over time [9]. Organ function deteriorates with age, which can cause hearing loss, blurred vision, mental deterioration, muscle deterioration, and other effects. These health problems are related to geriatric syndrome, namely, frailty, urinary incontinence, delirium, falls, and pressure ulcers, which become predictors of disease and death [10].

## 2. Oxidative Stress and Inflammation in Ageing and Degenerative Diseases

**Figure 1:**
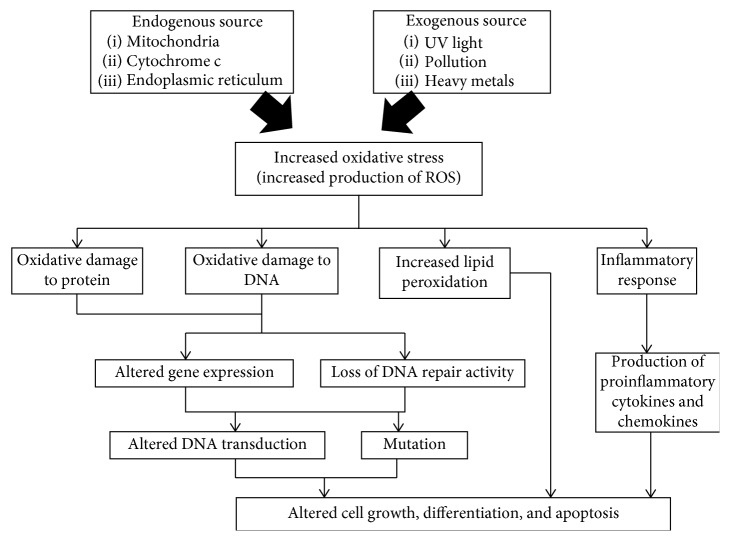
Oxidative stress and inflammation in ageing and degenerative diseases [11–21].

Oxidative stress and inflammation are common factors that contribute to ageing and the development of degenerative diseases. Oxidative stress occurs when there is an imbalance between reactive oxygen species (ROS) and antioxidants [11]. ROS are derived from endogenous sources such as mitochondria, cytochrome c, and the endoplasmic reticulum and exogenous sources such as UV light, pollution, and heavy metals [12]. ROS, including hydrogen peroxide (H_2_O_2_), hydroxyl radical (.OH), nitric oxide (NO), and superoxide anion (O^2–^), can diffuse through cell membranes, resulting in cellular oxidative metabolism [13]. Excessive production of ROS in the body causes oxidative damage to several types of biological molecules, such as DNA, carbohydrates, protein, and lipid (Figure 1). The oxidative damage to protein and DNA could result in altered DNA transcription and the loss of DNA repair capacity [14]. Moreover, oxidative damage to lipids leads to an increase in lipid peroxidation [15]. The accumulation of this molecular damage could cause mitochondrial dysfunction, induce mutations, and alter cell growth, differentiation, and apoptosis. Apoptosis or programmed cell death involves two apoptotic pathways, namely, the intrinsic and extrinsic pathways [22]. During the process of apoptosis, cytochrome c conjugates with procaspase-9. Apaf-1 is released and forms an apoptosome complex. This complex induces the activation of caspase-9 and further activates caspase-3 before inducing the apoptosis pathway [22].

In addition, the production of ROS is essential in signalling pathways and in the mediation of inflammation [16]. A previous study reported that ageing and degenerative diseases are also associated with the presence of low-grade persistent inflammation [23]. Under normal conditions, inflammation is important for the injury-repair cycle and primary defence against pathogens, which works efficiently with minimal secondary damage [24]. However, during the ageing process, the sensitivity to the molecular response and receptor expression levels are changed due to the long-term and repeated stimulus-response cycles. The transcription factor nuclear factor-κB (NF-κB) is one of the major factors that integrate the intracellular regulation of inflammation, and the dysregulation of this pathway is associated with age-related disease and ageing [25]. The dysregulation of NF-κB will lead to inflammation and disease development. Under normal conditions, NF-κB is localized in the cytoplasm in the form of a heterodimer. However, in response to oxidative stress, inflammatory signals such as TNF-*α* and LPS induce the phosphorylation of the I-κB proteins in NF-κB, resulting in the activation and subsequent nuclear translocation of NF-κB [17]. The translocation of NF-κB activates target genes that could contribute to cellular senescence, apoptotic signalling, senescence-associated secretory phenotype (SASP), and the production of other proinflammatory cytokines, such as IL-1*β*, leading to the development of disease [18]. A previous study showed that the expression levels of NF-κB and inflammatory genes were higher in human fibroblasts derived from the skin of older individuals and patients with HGPS progeria than in those derived from young individuals [19]. In addition, a previous study also showed that older people typically have higher levels of interleukin-6 (IL-6), C-reactive protein (CRP), and TNF-*α* in their blood [26]. This finding was supported by another study investigating sarcopenia, which reported that the inflammatory cytokines IL-6, IL-1*β*, and TNF-*α* weakened the anabolic signalling cascade, leading to sarcopenia [27]. These findings indicate the role of oxidative stress and inflammation in ageing and the development of degenerative diseases, especially, AD, PD, atherosclerosis, type 2 DM, and hypertension.

### 2.1. Ageing and Degenerative Disease

AD is the most common neurodegenerative disease that can cause dementia in elderly people. AD impacts the number of neurons in the brain, which is associated with learning and memory [28]. AD can be categorized into two types: late-onset sporadic AD and early-onset familial AD [29]. Commonly, late-onset sporadic AD affects individuals older than 65 years of age, while early-onset AD afflicts individuals younger than 65 years of age. Early-onset AD occurs due to genetic mutations in the genes encoding presenilin-1 (PS1), presenilin-2 (PS2), and amyloid precursor protein. AD pathologies can be evaluated by the existence of *β*-amyloid accumulation, oxidative stress, neurofibrillary tangles, and senile plaque and a reduction in the level of acetylcholine in the brain [30]. In a previous study, products of protein oxidation (protein carbonyl and 3-nitrotyrosine) and lipid peroxidation (malondialdehyde (MDA), 4-hydroxynonenal, and F2-isoprostanes) and oxidative stress markers were also shown to be increased in AD brains [21, 31, 32]. The accumulation of free radicals in the central nervous system and the peripheral tissue in the brain alters the activities and expression of antioxidant enzymes, resulting in the development of AD [21].

PD is the second most common neurodegenerative disease after AD [33]. The prevalence of this disease increases with age, and 1% of the population older than 60 years of age is diagnosed with PD [34]. Most individuals with PD manifest some signs and symptoms, including rest tremor, rigidity, postural instability, and slow movements. PD is characterized by the accumulation of a-synuclein protein within neurons inside Lewy neurites and Lewy bodies [35]. PD can be caused by hereditary and environmental factors, including oxidative stress and iron accumulation in the brain [33, 36]. According to Medeiros et al. [36], oxidative stress levels and inflammatory markers were significantly increased in PD patients. This finding was supported by another study carried out by Tosukhowong et al. [37], which reported that oxidative stress is the main factor that contributes to the pathogenesis of PD. The presence of oxidative stress and iron can cause harm to the brain structure, which leads to the death of dopaminergic neurons in the substantia nigra [36]. Thus, the loss of these dopaminergic neurons in the substantia nigra will lead to progressive motor impairment in PD [33, 36].

Atherosclerosis is also considered an ageing-related and degenerative disease that can be characterized by the accumulation of lipids and inflammatory cells in the lumen of the artery, resulting in the formation of plaques in the artery and leading to the interruption of blood flow [38]. Oxidative stress is one of the factors that contribute to the pathogenesis of atherosclerosis by inducing the oxidative modification of low-density lipoprotein (LDL) [39]. In atherosclerosis, angiotensin II, which acts as a vasoconstrictor, causes an imbalance between ROS and antioxidants in the vascular system by inducing the generation of ROS through the activation of NADPH oxidase [40]. Excess ROS production due to NADPH oxidase activation will lead to inflammation and inflammatory cytokine production through the activation of NF-κB [41]. Excess production of ROS also regulates atherosclerotic events, such as infiltration, migration, adhesion, and platelet activation, which can cause plaque formation in the artery.

Furthermore, type 2 DM is a complex chronic metabolic disease that can be caused by various factors, including obesity, the environment, and genetics [42]. Insulin resistance is a major risk factor that contributes to the development of type 2 DM among the elderly. Type 2 DM can lead to chronic complications such as end-stage kidney disease, cardiovascular disease, blindness, and limb amputations [42]. Type 2 DM is characterized by the presence of inflammation and a high oxidative stress level [43] because insulin resistance in type 2 DM is associated with decreased antioxidant capacity and increased free radicals, which leads to the oxidative damage of cellular components [44]. By affecting transcription factors, oxidative stress induces the development of insulin resistance and contributes to the activation of serine/threonine kinases and proinflammatory cytokines, including IL-6 and TNF-*α* [45]. The activation of serine/threonine kinases disturbs the cellular redistribution of insulin signalling components and decreases GLUT4 gene transcription, which can reduce glucose metabolism [45]. A reduction in glucose metabolism and the activation of inflammation will lead to the development of insulin resistance.

Hypertension is another example of an ageing-related and degenerative disease in elderly people. Hypertension is a risk factor for heart failure, stroke, myocardial infarction, peripheral arterial disease, and aortic aneurysm. Hypertension is associated with impaired endothelial function, which is related to the production of NO [46]. NO is released by the endothelium, which is important in controlling vascular relaxation. Hypertension occurs when the levels of catalase and superoxide dismutase are insufficient to respond to the level of oxidative stress [47]. Increased oxidative stress will cause a decrease in NO availability, which disrupts vascular tension. In addition, hypertension is also related to angiotensin-I-converting enzyme (ACE), which is involved in vascular tension [48]. ACE as a vasoconstrictor is essential for the conversion of angiotensin I into angiotensin II and stimulates aldosterone secretion. ACE activation indirectly increases blood pressure by constricting the blood vessel.

As oxidative stress and inflammation play an important role in the pathogenesis of various types of disease, the introduction of antioxidant and anti-inflammatory agents, such as ginger, can show potential to greatly impact the prevention and treatment of these diseases.

## 3. Ginger (*Zingiber officinale* Roscoe)

**Figure 2:**
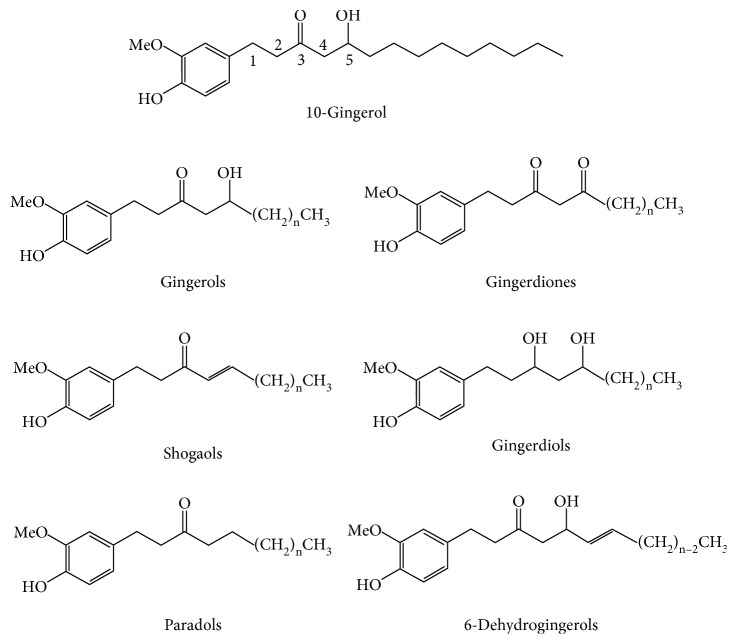
Chemical structures of some active compounds in ginger, *Zingiber officinale* Roscoe [55].

Ginger has been studied as an antiageing agent that protects against oxidative stress and inflammation in the pathogenesis of degenerative disease and ageing. Ginger (*Z*. *officinale* Roscoe) is considered a traditional herb; it is used not only as a spice or flavour [49] in cooking but also as a traditional medicine to treat various types of health problems such as diabetes [50], nausea [51], migraine [52], and others. Commonly, ginger can be found in subtropical and tropical Asia, Africa, Far East Asia, China, and India [53]. Ginger is composed of several bioactive compounds, including 6-gingerol, 6-shogaol, 10-gingerol, gingerdiones, gingerdiols, paradols, 6-dehydrogingerols, 5-acetoxy-6-gingerol, 3,5-diacetoxy-6-gingerdioal, and 12-gingerol, that contribute to many biological activities of ginger [53–55]. However, the primary active compounds in ginger are gingerol and shogaol [53].  Figure 2 shows the chemical structures of several active compounds in ginger.

Due to its bioactive compounds and constituents, ginger has shown various types of therapeutic effects, including antibacterial [54, 56–60], anticancer [61, 62], anti-inflammatory [63], antidiabetic [64, 65], gastroprotective [65], antioxidant [66, 67], and neuroprotective activities [68].

### 3.1. Antibacterial Properties

A previous study performed by Sebiomo et al. [69] found that ginger extract exhibits antibacterial activities against Gram-positive bacteria, including *Staphylococcus aureus* and *Streptococcus pyogenes* [69]. This result was supported by another study that observed the antibacterial activity of an ethanolic ginger extract against *Escherichia coli* and *Salmonella typhi* [59]. Ginger also exhibited antibacterial effects against the Gram-positive bacteria *Enterococcus faecalis* [70]. In that study, the antiadhesion activity of ginger oil was greater than that of *Orthosiphon stamineus* extract in a suspension of *E*. *faecalis*. Another study carried out by Chakotiya et al. [56] confirmed the antibacterial properties in ginger. These researchers reported that ginger effectively inhibited the growth of *Pseudomonas aeruginosa*, a bacterium that can form biofilms in the human body. In a different study, the significant antibacterial effect of an ethanol extract of ginger root was higher than that of an ethanol extract of ginger leaf and a water extract of ginger root [69]. Most of the antibacterial activity of ginger extract is dose-dependent and depends on the type of extraction [58].

### 3.2. Antioxidant Properties

In addition, ginger is also a good source of antioxidants and shows high antioxidant activity following alcohol extraction [67]. It has been shown that extracts prepared with the solvents methanol and ethanol showed higher free radical scavenging and reducing power activities than did extracts prepared with water. A previous study conducted by Maizura et al. [71] reported that the activity of ginger in DPPH radical scavenging and FRAP assays was higher than that of turmeric extract [71]. However, the antioxidant activity of ginger extract was lower than that of kesum extract. Another study found that the antioxidant activity of 10-gingerol and 6-shogaol was higher than that of 6-gingerol and 8-gingerol at 60°C [66]. These antioxidant properties were indicated by the presence of hydroxyl groups and solubilizing side chains in the chemical structure of the active compound [66]. A study conducted by Yusof and Abdul-Aziz [72] reported that ginger extract has great potential to function as an antioxidant such as superoxide dismutase (SOD), glutathione peroxidase (GPx), and catalase (CAT) in the elimination of accumulated free radicals (superoxide radicals and hydrogen peroxide) in a hepatoma cell line [72]. Compared with the control condition, treatment with ginger extract at a concentration of 200–500 *μ*g/ml caused a significant reduction in SOD, GPx, and CAT activities in a hepatoma cell line. In addition, the antioxidant properties of ginger have also been proven to reduce insulin resistance in diabetes by enhancing glucose transport activity and improving glucose tolerance [73]. Another study found that ginger can act as an antidiabetic agent by decreasing cholesterol, serum glucose, and triacylglycerol [64]. In diabetic rats, ginger has been shown to reduce urine protein levels and to cause hypoglycaemia, hypocholesterolaemia, and hypolipidaemia [64].

### 3.3. Anti-Inflammatory Properties

Ginger has been reported as an anti-inflammatory agent by inhibiting cyclooxygenase-2 (COX-2) and decreasing the production of inflammatory factors, TNF-*α* and IL-*β* [55, 74]. This finding was supported by another study that mentioned that ginger has the ability to reduce the levels of TNF-*α* and hs-C-reactive protein (hs-CRP) in patients with diabetes [73]. Ginger extract can act synergistically with antituberculosis therapy by reducing TNF-α levels, lipid peroxidation, and MDA in patients with tuberculosis [75].

### 3.4. Neuroprotective Properties

Hussein et al. [68] also reported that due to the presence of polyphenolic compounds, ginger has potential to be a neuroprotective agent that can reduce the neurotoxic effect of MSG by altering neurotransmitter levels and suppressing 8-hydroxy-2′-deoxyguanosine (8-OHdg) and amyloid accumulation [68]. This study also reported that ginger improves the histological features of the brain and attributed this effect to the antioxidant properties of ginger [68]. A previous study reported that ginger displayed a protective role in the brain of people with diabetes by reducing oxidative stress, inflammation, and apoptosis [76]. This study also revealed that ginger reduced Ach expression, modulated the astroglial response to injury, and improved neurogenesis. In a different study investigating diabetic rats, the neuroprotective property of ginger was revealed by the decrease in MDA level and the acceleration of the brain-oxidant defence mechanism [77]. The accelerated brain-oxidant defence was indicated by the activities of SOD, CAT, and GPx, which were significantly different from the normal level.

### 3.5. Anticancer Properties

In addition, the active compounds in ginger, including 6-gingerol and 6-shogaol, have been shown to have anticancer properties by inhibiting COX-2 expression [78], suppressing NF-*β* DNA binding activity [78], and enhancing BAX expression [79]. The anticancer properties of ginger are also evidenced by the inhibition of oval cell proliferation and caspase-8 expression, which is essential for inducing apoptosis and downregulating the Bcl-2 protein [80]. A previous study also reported that ginger extract enhanced the anticancer effects of 5-FU against colorectal cancer [81]. In that study, compared with treatment with 5-FU alone, treatment with ginger extract resulted in increased apoptosis. The anticancer effect of 5-FU significantly increased with the combination of ginger extract and Gelam honey.

## 4. Role of Ginger in Oxidative Metabolism

Recent studies have shown that ginger effectively protects against ROS. Ginger extract has been found to reduce the production of ROS and the level of MDA, which is related to lipid peroxidation [82, 83]. Another study found that the introduction of ginger to a human chondrocyte cell model, with oxidative stress induced by interleukin-1, decreased the production of ROS and lipid peroxidation and induced the expression of antioxidant enzymes, including CAT, SOD1, GPx1, GPx3, and GPx4 [84]. This result is consistent with the finding of another study, which showed that the treatment of diabetic rats with ginger increased GSH level and SOD, CAT, GR, and GPx activities and decreased the MDA level [85], indicating the restoration of antioxidant enzymes. The antioxidant and anti-inflammatory activities of ginger extract could also be seen through the reduction in caspase-3 activation and the ratio of Bax/Bcl-2 by blocking IL-1*β* [22, 86]. The reduction in caspase-3 activation could inhibit the signalling of the apoptosis pathway, which leads to the pathogenesis of the disease. This finding is supported by previous reports showing that ginger extract inhibited the activation of caspase-8, caspase-3, and caspase-9 in ecstasy-induced neurotoxicity [87].

Additionally, bioactive compounds in ginger extract, including 6-shogaol, displayed antioxidant properties through the nuclear factor erythroid 2-related factor 2 (Nrf2) signalling pathway [88]. Nrf2 is a transcription factor that regulates the expression of the cytoprotective molecules in oxidative stress and protects multiple organs and cells [89]. Under normal conditions, Nrf2 is connected to its inhibitory partner Kelch-like ECH-associated protein 1 (Keap1) in the cytosol [90, 91]. The introduction of ginger causes the dissociation of Nrf2 from Keap1 in the cytosol, and Nrf2 subsequently translocates into the nucleus, where Nrf2 binds to antioxidant response element (ARE) and initiates the transcription of antioxidant genes such as thioredoxin 1, thioredoxin reductase 1, and heme oxygenase-1, which could result in a protective effect on ageing and degenerative diseases [91].

## 5. Ginger as an Antioxidant Involved in Delaying Ageing

Since oxidative stress contributes to the pathogenesis of ageing and degenerative diseases, ginger has been widely studied with *in vitro*, *in vivo*, and human studies. Several reports have documented the effect of ginger as an antioxidant on delaying the ageing of several organs. Ilkhanizadeh et al. [92] reported that the antioxidant properties of ginger extract significantly decreased structural heart abnormalities in diabetic rats by improving the levels of serum apolipoproteins, leptin, homocysteine (Hcy), and cathepsin G. Cathepsin G is responsible for inducing necrosis and myocyte hypertrophy as well as increasing fibrosis through the conversion of angiotensin I to angiotensin II [93]. The antioxidant effects of the active compounds of ginger, specifically gingerol and shogaol, on heart structure can also be seen through the inhibition of leukotriene and prostaglandin biosynthesis by suppressing 5-lipooxygenase synthetase [94]. This finding was similar to the finding from a study by Shirpoor et al. [95], which showed that the lung abnormalities induced by oxidative stress can be improved by the antioxidant and anti-inflammatory properties of ginger.

In addition to the protective effect on the heart, ginger also exhibits neuroprotective properties through the restoration of structural and morphological brain damage caused by diabetes. El-Akabawy and El-Kholy [76] found that the administration of ginger extract (500 mg/kg) to diabetes-induced rats improved structural and morphological changes related to diabetes. This protective role of ginger is due to decreased oxidative stress, apoptosis, inflammation, astroglial response to injury, and acetylcholinesterase expression [76]. These changes can be seen in different regions of the brain in diabetes, namely, the frontal cortex, dentate gyrus, and cerebellum. The neuroprotective properties of ginger were confirmed by a previous study [96]. It was found that the oral administration of ginger extract to mice with SCO-induced memory loss upregulated the level of brain-derived neurotrophic factor (BDNF), which is essential to neuronal maintenance and survival, synaptic plasticity, and cognitive processes. This study suggested that ginger extract may have a potential effect on the management of memory loss in patients with amnesia and AD.

The effect of the high antioxidant activity of ginger on cognitive function has been demonstrated in numerous studies. In human studies, ginger has been shown to affect the cognitive function of elderly people [97]. Supplementation with 400 mg and 800 mg of ginger for two months enhanced cognitive processing and attention in middle-aged women without side effects [97]. This finding was supported by another study that showed ginger supplementation for three months has potential to enhance cognition in postmenopausal women by improving the continuity of attention, the power of attention, speed, and the quality of memory [98]. This effect occurs due to the effects of the active compounds in ginger, which inhibit the cholinesterase activity, resulting in an increased level of acetylcholine that is essential for learning and memory processing [99]. Improved cognitive function can also be seen with *in vivo* studies in animal models. The oral administration of ginger extract (100 and 200 mg/kg) to Wistar rats improved the brain impairment induced by morphine [100]. The results showed that the total time spent in the dark compartment was lower in the group treated with ginger extract than in the control group. This result was supported by a previous finding that the administration of the ginger extract at doses of 100 and 200 mg/kg improved cognitive function and neuronal density in the hippocampal regions of rats [101]. Moreover, the infarct volume of rats was decreased. This study demonstrated that the antioxidant activity of ginger can enhance cognitive function and exert neuroprotective effects in rats.

## 6. Ginger in the Prevention and Treatment of Degenerative Disease

In addition to delaying ageing, ginger has also been shown to prevent and treat several degenerative diseases. The phenolic contents in ginger with acetylcholinesterase enzyme inhibitory activities and antioxidant activity were investigated in AD [30]. The findings of this study showed that ginger extract has a high capacity to scavenge free radicals in the DPPH assay, indicating its antioxidant and antiacetylcholinesterase activity. Ginger extract also inhibited butyrylcholinesterase and increased cell survival against *β*-amyloid, which can induce toxicity in neuronal cells [30]. This result was confirmed by a previous finding that 6-gingerol effectively suppresses the expression of *β*-amyloid, which is induced by the accumulation of ROS and nitrogen species, increases the expression of antioxidant enzymes, and restores glutathione levels [102]. However, in an *in vivo* study using a rat model of AD, Zeng et al. [103] reported that ginger, which contains gingerol, improved learning and memory and reduced oxidative stress and inflammation. The results of this study suggested that a high dose of ginger extract increased the number of Nissl bodies and neurons, increased the activation of superoxide dismutase (SOD) and catalase (CAT), and decreased the levels of MDA, NF-κB, and IL-1. Another study demonstrated the ability of ginger to act as a protective and therapeutic agent in AD rats [104]. These researchers reported that compared with untreated AD rats, AD rats treated with 108 or 216 mg/kg of ginger exhibited significantly improved activity and acetylcholine level, significantly improved T-maze test results, and reduced acetylcholinesterase activity. The histopathological findings showed that after ginger consumption, amyloid plaques in AD rats disappeared [104].

In PD, the administration of 6-shogaol significantly reduced astrogliosis and microgliosis in the brain of a PD mouse model and enhanced the expression of nerve growth factor (NGF) and synaptic molecules in the brain [105]. This finding suggests that the active compound in ginger may reduce cognitive dysfunction in PD by inhibiting the inflammatory response, increasing the NGF level, and improving the formation of synapses in the brain with AD. Park et al. [106] reported that 6-shogaol protected dopaminergic neurons against MPTP- and MPP^+^-induced neurotoxicity in an *in vitro* and *in vivo* PD model. This protection of dopaminergic neurons can be seen through the inhibition of the inflammatory pathway, including TNF-*α*, NO, COX-2, and inducible nitric oxide synthase (iNOS), in the substantia nigra pars compacta and in the stratum. This finding was similar to the result from another study carried out by Ha et al. [107].

Additionally, in diabetes, ginger has exhibited strong antioxidant activities and inhibitory activities against enzymes linked to type 2 DM, specifically *α*-amylase and *α*-glucosidase [108]. *α*-Amylase is an essential enzyme that degrades complex dietary saccharides into oligosaccharides and disaccharides before their conversion into monosaccharides by *α*-glucosidase. The overexpression of these two enzymes can cause hyperglycaemia. In a previous study carried out by Akinyemi et al. [109], it was illustrated that ginger displayed antihypercholesteraemic properties in rats fed a high cholesterol diet. This antihypercholesteraemic property is exhibited by the ability of ginger to inhibit ACE. In a human study, ginger supplementation for eight weeks affected insulin levels and resistance and the lipid profile, namely, triglycerides (TG) and low-density lipoproteins (LDL), in patients with type 2 diabetes [73]. Ginger supplementation could reduce insulin, TG, and LDL-C levels in diabetic subjects. However, another study found that ginger decreased TG and serum total cholesterol levels in diabetic patients but showed no effect on LDL and high-density lipoprotein (HDL) levels [50]. The reduction in serum cholesterol concentration after ginger supplementation is due to the increase in hepatic cholesterol hydroxylase enzyme activity, which is important for the conversion of cholesterol into bile acids [50]. In another study, supplementation with 3 g/day of ginger for three months decreased glucose, insulin resistance, MDA, and CRP levels and significantly improved total antioxidant capacity (TAC) and paraoxonase 1 (PON-1) in patients with diabetes [43]. The improvement of TAC confirmed that ginger can act as an antioxidant agent to reduce oxidative stress and lipid peroxidation [43].

Moreover, ginger extract is also considered an effective anti-inflammatory agent in preventing osteoarthritis and rheumatoid arthritis [110]. In a human study of osteoarthritis disease, the consumption of 1 g/d ginger was found to decrease two inflammatory factors, TNF-*α* and IL-*β*, which can cause the activation of the lipoxygenase (LX) pathway and induce the nitric oxide synthase (iNOS2)/cyclooxygenase-2 (COX-2) pathway [74]. Another study showed that after three months of ginger supplementation, the serum concentrations of NO and hs-CRP were decreased in patients with osteoarthritis [111]. This finding could be due to the decreased activation of nitric oxide synthase and the increased systemic response to inflammatory events. Ginger not only decreases the level of inflammatory cytokines in osteoarthritis but also acts as an effective therapeutic agent in reducing stiffness, pain, and difficulty in patients with knee osteoarthritis [112]. The reduction in pain was assessed using a visual analogue scale (VAS).

On the other hand, ginger extract has shown a protective effect on the development of cardiovascular diseases such as coronary atherosclerosis and hypertension. According to a previous study, the infarct size in coronary atherosclerotic rabbits was reduced after 75 days of consuming ginger extract [113]. The total serum cholesterol in atherosclerotic rabbits also decreased. Another study showed that dietary consumption of ginger extract decreased the development of atherosclerotic lesions in rats [114]. This result was associated with a reduction in plasma LDL cholesterol level, LDL atherogenic modification, and the oxidative response of macrophages. This finding was confirmed by a previous study that showed the ability of ginger extract to reduce atherosclerotic lesions in the artery and to reverse the inflammatory cytokine expression and lipid profile induced in mice with atherosclerosis [115]. Another previous *in vitro* study demonstrated that ginger crude extract induced the relaxation of porcine coronary arteries in an endothelium-dependent manner [116]. Ginger extract was also vasoprotective in coronary arteries through the suppression of the cyclooxygenase pathway and nitric oxide synthase.

In addition, an *in vivo* study using Wistar rats showed that ginger extract decreased the level of lipid and blood pressure in hypertensive and hyperlipidaemic rats [117]. Another *in vitro* study supported the finding that ginger extract inhibited ACE activity in a dose-dependent manner [109]. This result was observed because the ACE produced by renin is involved in cleaving angiotensin I into angiotensin II, a vasoconstrictor that becomes a significant factor in hypertension. In a human study, ginger intake caused a significant reduction in the blood pressure of patients with hypertension and coronary heart disease [118]. This study demonstrated that the risk of hypertension and coronary heart disease was significantly decreased to 8% and 13% by consuming 1 gram of ginger per day. This effect is due to the antihypertensive properties of ginger, which regulate the inhibition of ACE and prevent lipid peroxidation in the heart [83]. Thus, ginger can be used as an alternative therapy in the prevention of ageing and degenerative diseases. Current research findings showing the effects of ginger on ageing and degenerative diseases from *in vitro* studies are summarized in Table 1, while *in vivo* studies are shown in Table 2, and human studies are shown in Table 3.

## 7. Conclusion and Perspectives

In this review, we discussed the current evidence on the potential role of ginger and its active compounds in the prevention of ageing and degenerative diseases. Ageing and degenerative diseases are geriatric syndromes characterized by the progressive loss of physiological function, which leads to unfavourable consequences, including morbidity and mortality. Understanding the major risk factors of these diseases is imperative to find ways to delay and prevent these diseases. A previous study has shown that continuous exposure to oxidative stress could lead to an increase in ROS production and induce inflammation, which could result in damage to several molecules, including DNA, protein, and lipid [23].

As oxidative stress and inflammation contribute to the pathogenesis of ageing and degenerative diseases, ginger (*Z*. *officinale* Roscoe) has been used as an antiageing agent. Ginger and its active compounds, including 6-gingerol, 6-shogaol, 10-gingerol, gingerdiones, gingerdiols, and paradols, exhibited antiageing effects in various types of age-related and degenerative diseases through their antioxidant and anti-inflammatory properties [53]. The antioxidant and anti-inflammatory properties of ginger could reduce the level of oxidative stress and inflammation markers by counteracting the production of ROS [55]. Many studies have proven that ginger can reduce the levels of MDA, TNF-*α*, IL-1*β*, and CRP and that ginger can be applied as an antiageing agent.

However, the current review investigating the effect of ginger is only limited to certain types of age-related and degenerative diseases. Until now, no study has discussed the effect of ginger on muscular diseases, such as sarcopenia and muscular dystrophy, which have become a major concern for elderly people. In addition, studies on the effective dosage, pharmacodynamics, and pharmacokinetics of ginger, which can benefit the prevention of ageing and degenerative diseases, are still inadequate. Hence, additional studies on ginger need to be conducted to increase our understanding of the role and mechanism of ginger in the prevention of disease.

## Figures and Tables

**Table 1:** Effect of ginger on degenerative disease (*in vitro* studies).

Related disease	Constituent	Effects	References
Alzheimer's disease	Ginger root extract	(i) Showed antioxidant and antiacetylcholinesterase activity (ii) Inhibitory effects towards butyrylcholinesterase (iii) Increased cell survival following *β*-amyloid expression.	Tung et al. [30]
	6-Gingerol	(i) Suppressed the expression of *β*-amyloid (ii) Increased the expression of antioxidant enzyme (iii) Restored glutathione level	Lee et al. [102]

Parkinson's disease	6-Shogaol	(i) Protected dopaminergic neurons against MPTP- and MPP^+^-induced neurotoxicity	Park et al. [106]
	6-Shogaol	(i) Inhibited the release of NO and the expression of inducible nitric oxide synthase (iNOS)	Ha et al. [107]

Type 2 diabetes mellitus	Ginger extract	(i) Exhibited strong antioxidant activities	Oboh et al. [108]

Cardiovascular disease	Ginger extract	(i) Induced the relaxation of coronary arteries (ii) Increased vasoprotection through the suppression of the cyclooxygenase pathway and nitric oxide synthase	Wu et al. [116]

**Table 2:** Effect of ginger on degenerative disease (*in vivo* studies).

Related Disease	Constituent	Effects	References
Alzheimer's disease	Ginger root extract	(i) Improved learning and memory (ii) Increased the number of Nissl bodies and neurons (iii) Increased the activation of SOD and CAT (iv) Decreased the levels of MDA, NF-κB, and interleukin-1 (IL-1)	Zeng et al. [103]
	Aqueous ginger infusion	(i) Improved the activity and level of acetylcholine (ii) Improved T-maze test results and reduced acetylcholinesterase activity (iii) Induced the disappearance of amyloid plaques	Karam et al. [104]

Parkinson's disease	6-Shogaol	(i) Reduced astrogliosis and microgliosis in the brain (ii) Enhanced the expression of nerve growth factor (NGF) level and synaptic molecules (iii) Inhibited the inflammatory response	Moon et al. [105]
	6-Shogaol	(i) Improved the formation of synapses in the brain (ii) Inhibited components of the inflammatory pathway such as TNF-*α*, NO, COX-2, and inducible nitric oxide synthase (iNOS)	Park et al. [106]

Type 2 diabetes mellitus	Fresh ginger sample	(i) Exhibited inhibitory activities against *α*-amylase and *α*-glucosidase (ii) Inhibited ACE activity	Akinyemi et al. [109]

Cardiovascular disease	Ginger extract	(i) Reduced infarct size (ii) Reduced total cholesterol serum	Rouhi-Boroujeni et al. [113]
	Ethanolic ginger extract	(i) Decreased the development of atherosclerotic lesions (ii) Reduced plasma levels, LDL cholesterol levels, LDL atherogenic modifications, and the oxidative response of macrophages	Fuhrman et al. [114]
	6-Gingerol	(i) Reduced atherosclerotic lesions in arteries (ii) Reversed the expression of inflammatory cytokines and lipids	Wang et al. [115]
	Dried ginger powder	(i) Decreased lipid levels and blood pressure	Sanghal et al. [117]
	Aqueous ginger extract	(i) Inhibited ACE activity (ii) Prevented lipid peroxidation in the heart	Akinyemi et al. [83]
	Fresh ginger sample	(i) Inhibited ACE activity	Akinyemi et al. [109]

**Table 3:** Effect of ginger on degenerative disease (human studies).

Related disease	Constituent	Effects	References
Type 2 diabetes mellitus	Fresh ginger rhizomes	(i) Reduced the levels of triglycerides (TG) and low-density lipoprotein (LDL) (ii) Reduced insulin	Mahluji et al. [73]
	Powdered ginger rhizomes	(i) Decreased TG level and total serum cholesterol (ii) No effect on LDL and high-density lipoprotein (HDL) (iii) Increased activity of hepatic cholesterol hydroxylase enzymes	Arablou et al. [50]
	Powdered ginger capsule	(i) Decreased the levels of glucose, malondialdehyde (MDA), and C-reactive protein (CRP) and insulin resistance (ii) Improved total antioxidant capacity (TAC) and serum paraoxonase-1 (PON-1)	Shidfar et al. [43]

Osteoarthritis	Powdered ginger capsule	(i) Decreased the level of tumour necrosis alpha (TNF-*α*) and interleukin-beta (IL-*β*)	Mozaffari-khosravi et al. [74]
	Powdered ginger capsule	(i) Reduced the level of nitrite oxide (NO) (ii) Reduced hs-C-reactive protein (hs-CRP)	Naderi et al. [111]
	Powdered ginger capsule	(i) Reduced stiffness, pain, and difficulty in patients with knee osteoarthritis	Zakeri et al. [112]

Cardiovascular disease	Powdered ginger capsule	(i) Reduced blood pressure in patients with hypertension and coronary heart disease	Wang et al. [118]
